# Enhancing skill conceptualization, critical thinking, and nursing knowledge through reflective case discussions: a systematic review

**DOI:** 10.25122/jml-2023-0042

**Published:** 2023-06

**Authors:** Haeril Amir, Pande Ayu Naya Kasih Permatananda, Desy Dwi Cahyani, Wahyuny Langelo, Rosita Rosita, Sajodin Sajodin, Richa Noprianty, Anggia Astuti, Suhari Suhari, Sri Wahyuningsih, Prima Dewi Kusumawati, Prita Dhyani Swamilaksita, Sudarman Sudarman, Syaiful Syaiful

**Affiliations:** 1 Faculty of Public Health, Universitas Muslim Indonesia, Makassar, Indonesia; 2 Faculty of Medicine and Health Science, Universitas Warmadewa, Bali, Indonesia; 3 Midwifery Department, Poltekkes Kemenkes Malang, Malang, Indonesia; 4 Universitas Katolik De La Salle, Manado, Indonesia; 5 Nursing Study Program, Akademi Keperawatan Justitia, Palu, Indonesia; 6 Faculty of Health Science, Universitas Aisyiyah Bandung, Bandung, Indonesia; 7 Bachelor of Applied Nursing Anesthesiology, Bhakti Kencana University, Bandung, Indonesia; 8 Faculty of Nursing, Universitas Jember, Jember, Indonesia; 9 Faculty of Nursing, Institut Ilmu Kesehatan Strada Indonesia, Kediri, Indonesia; 10 Faculty of Health Science, Universitas Esa Unggul, Jakarta, Indonesia; 11 Universitas Megarezky, Makassar, Indonesia

**Keywords:** reflective case discussion (RCD), nurse, professional, systematic review

## Abstract

Reflective case discussion (RCD) is a reflective activity conducted by nurses, midwives, and other healthcare workers to enhance their skills, critical thinking, and knowledge. This systematic review follows the PRISMA Guideline checklist and includes articles from various databases, such as Scopus, PubMed, ProQuest, and ScienceDirect. The quality assessment of each article was performed using the Critical Appraisal Skills Program (CASP). During the initial database search, we retrieved 997 articles from Scopus, 700 articles from ProQuest, 357,554 articles from PubMed, and 1,526 articles from ScienceDirect. The search was conducted using relevant keywords, including "reflective case discussion," "nursing," "critical thinking," "skills," and "knowledge." Following the inclusion and exclusion criteria, eight relevant articles were identified, excluding duplicate studies, limited to full papers, open access, conducted in a hospital setting, and written in English. The findings demonstrate that RCD effectively enhances nurses' skills, critical thinking, and knowledge, contributing to their professionalism in patient care. RCD also proved beneficial in preventing repetitive mistakes and promoting teamwork among nurses. Thus, RCD should be embraced as a valuable form of Continuing Professional Development (CPD) and integrated into nurses' ongoing learning processes.

## INTRODUCTION

Reflective practice is an ongoing effort to develop the professional development and knowledge advancement of nurses worldwide [1, 2]. It has become an integral aspect of the nursing profession [3], as emphasized by Dewey, who considered it a form of active, persistent, careful consideration of assumptions or beliefs based on the reasons and conclusions that support them [4]. Nurses, as humans, use a reflective way in every action even though they are usually unaware of it [5].

The British Association of Sport and Exercise Sciences describes the reflective practice as allowing nurses to reflect on ideas and experiences as a team with group methods fostering relational approaches [6]. In other words, reflective practice cannot be separated from learning experiences [7] and becomes part of continuing professional development (CPD) [8].

Reflective practice, mainly conducted through reflective case discussions (RCD), is related to the professional attitude in delivering nursing care [8]. It encompasses the knowledge, skills, and critical thinking abilities required to analyze and address healthcare challenges. The significance of RCD is evident in various regulations and codes of conduct. For instance, RCD is stipulated in the Nursing and Midwifery Council's code of conduct in England, which outlines professional standards of practice and behavior for nurses [9]. Similarly, in Indonesia, it is stipulated by the Minister of Health of the Republic of Indonesia number 836/MENKES/SK/VI/2005 concerning guidelines for developing performance management for nurses and midwives [10]. RCD is carried out at least once a month [11]. However, implementing RCD in hospitals does not always run optimally, and there are many obstacles, such as limited leadership support from nursing managers and nurses' self-reflective abilities [12]. It should be noted that the main purpose of reflective practice is to bridge the gap between theory and practice in health services to produce professional services for patients [13]. Nevertheless, obstacles such as time constraints, heavy workloads [14], and suboptimal support from hospital administrators [8] can hinder its implementation. This literature review aimed to assess the influence of RCD on enhancing the critical thinking skills, knowledge, and abilities of nurses. The findings could provide valuable insights and recommendations for hospital management to promote the regular implementation, supervision, and evaluation of RCD in healthcare settings. Collaboration among all stakeholders is crucial in supporting the effective implementation of RCD at the hospital level.

## MATERIAL AND METHODS

This systematic review adhered to the Preferred Reporting Items for Systematic Reviews and Meta-Analysis Guideline (PRISMA) 2020 checklist [15]. We used the PRISMA guidelines as used in several previous review articles. The search strategy was designed to identify relevant literature based on the research objectives and predefined inclusion and exclusion criteria. PubMed, ScienceDirect, Scopus, and ProQuest were defined as the selected databases for the search.

In the PubMed database, the search was conducted using the keywords "case reflection discussion" [Mesh] and "nursing" [Mesh], resulting in 434 articles. Additionally, the keywords "critical thinking, skill, knowledge" [Mesh] and "nursing" were used, yielding 10,768 articles. Furthermore, a search using the ScienceDirect database with the first keyword "case reflection discussion" or "reflective practice and nursing" identified 12,981 articles. Using the second keyword, "critical thinking, skill, knowledge, and nursing," 17,052 articles were found. When these two keywords were combined, 4,281 articles were identified. Filtering the articles for the last 10 years resulted in 1,526 research articles.

An article search using the ProQuest database with the keywords "reflective practice" and nursing" found 31,709 articles. The second keyword, "critical thinking, skill, knowledge, and nursing," found 818,212 articles. If the first and second keywords were combined, 791,532 articles were obtained. After applying filters for full-text availability, open access, being in the English language, and published within the last 10 years, approximately 700 articles remained. Lastly, a search using the Scopus database with the keywords "reflective OR reflection AND nursing AND critical thinking AND professional" identified 997 articles.

**Figure 1:**
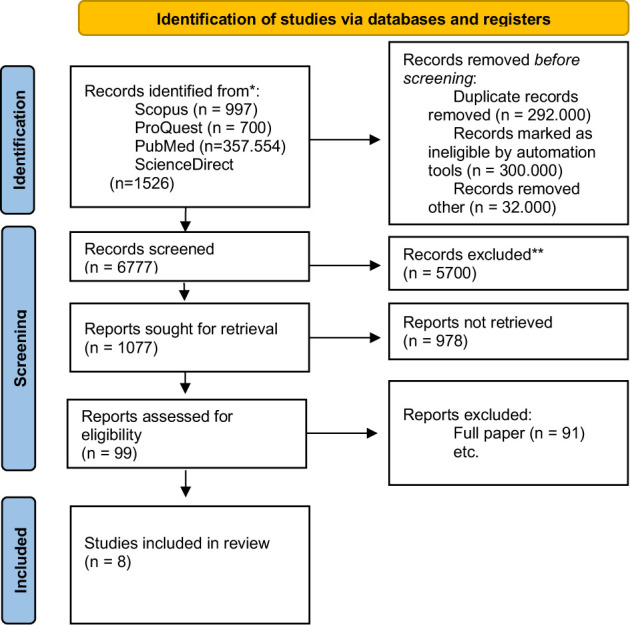
Flowchart PRISMA Diagram

## RESULTS

The initial search resulted in a number of titles from different databases. However, only 8 final articles were found eligible and used as references in this systematic review after undergoing a rigorous selection procedure based on inclusion and exclusion criteria and applying additional filters such as removing duplicate publications (Table 1).

**Table 1:** Synthetic Grid

Author	Title	Method	Population	Result and Conclusion
Ardian *et al*. [16 ]	Correlation between implementation case reflection discussion based on the Graham Gibbs Cycle and nurses’ critical thinking skills	Pre- and post-test without a control group	Nurses in inpatient rooms from a hospital (n=85 nurses)	Significant increase in nurses’ ability to think critically and components of engagement, cognitive maturity, and innovativeness after implementing CRD based on Graham Gibbs Cycle (p<0.05).
Madison *et al*. [17]	Reflective practice groups and nurse professional quality of life	Cross-sectional, quantitative research methodology	184 Australian nurses from a regional teaching hospital	Significantly higher scores for personal and job resources of autonomy, self-efficacy, skill discretion, social support, and group cohesion.
Benjamin *et al*. [18]	Evaluating the impact of reflective practice groups for nurses in an acute hospital setting	Cross-sectional, quantitative research methodology	Overall 251 nurses	Reflective practice group attendance was significantly correlated with increased compassion satisfaction
Dawber *et al*. [19]	A Longitudinal, Comparative Evaluation of Reflective Practice Groups for Nurses Working in Intensive Care and Oncology	Longitudinal, quantitative	Multidisciplinary team including 20 nurses	RPG facilitated them in increasing self-awareness, clinical insight, and quality of care.
Davis *et al*. [20]	Cultivating Clinical Judgment in Pharmacological Decision-Making Through Reflection on Practice	A nonrandomized repeated measures design	128 with intervention (n = 62) and control (n = 66) groups	Reflections on practice grew clinical judgment in decision making
Thomas *et al*. [21]	Archives of Psychiatric Nursing ‘ A different kind of space ’ : Mixed methods evaluation of facilitated reflective practice groups for nurses in an acute inpatient mental health unit	Mixed methods evaluation	114 nurses	Participants described the benefits gained from the reflection that took place in a group. Participants expressed ideas about togetherness, teamwork, and peer support.
Clouder *et al*. [22]	Reflective practice and clinical supervision: an interprofessional perspective	Clinical supervision	Could not identify	RPG improved practice and accountability and promoted professional development.
Maria *et al*. [23]	How Reflective Practice Improves Nurses’ Critical Thinking Ability	The researcher designed the pilot questionnaire	n=34	Reflective practice improved critical thinking

The study by Ardian *et al*. [16] demonstrated a significant implication of reflective case discussions (RCD) in improving the nurses’ critical thinking skills according to the Graham Gibbs Cycle. This is crucial since nurses are expected to think rationally and accurately in providing their patients with measured and precise actions. The assessment of this study, using the Critical Appraisal Systematic Review Checklist, showed positive results (YES) for criteria related to focus, type of paper, the relevance of the included studies, study quality, reasonableness, overall results, the precision of results, applicability to the local population, consideration of important outcomes, and the balance of benefits, harms, and costs.

Another study [24] stated that conducting RCD within a group can enhance efficacy, autonomy, and skills. The assessment using the Critical Appraisal Systematic Review Checklist, with questions similar to those in Table 2, also resulted in positive results (YES) for all criteria. Additionally, Benjamin *et al*. [23] found that nurses who participated in RCD activities became more cohesive and developed a greater sense of compassion towards each other. Furthermore, the studies by Dawber and Davis [21, 30] supported the implementation of RCD. Thomas, Clouder, and Maria also demonstrated the perceived benefits of RCD among participants. RCD directly fosters their professionalism and cohesiveness [21-23].

**Table 2:** Critical Appraisal Systematic Review Checklist

No	Appraisal Checklist	Ardian *et al*. (Ardian *et al*., 2019)	Madison *et al*. (Sundgren *et al*., 2021)	Benjamin *et al*. (Davey *et al*., 2021)	Dawber (Dawber & Brien, 2013)	Davis (Davis & Wood, 2022)	Thomas *et al*. (Thomas & Isobel, 2019)	Clouder *et al*. (Clouder & Sellars, 2004)	Maria *et al*. (Cirocco, 2007)
1	Did the review address a clearly focused question?	Yes	Yes	Yes	Yes	Yes	Yes	Yes	Yes
2	Did the authors look for the right type of papers?	Yes	Yes	Yes	Yes	Yes	Yes	Yes	Yes
3	Do you think all the important, relevant studies were included?	Yes	Yes	Yes	Yes	Yes	Yes	Yes	Yes
4	Did the review’s authors do enough to assess quality of the included studies?	Yes	Yes	Yes	Yes	Yes	Yes	Yes	Yes
5	If the results of the review have been combined, was it reasonable to do so?	Yes	Yes	No	Yes	Yes	Yes	Yes	Yes
6	What are the overall results of the review?	Yes	Yes	Yes	Yes	Yes	Yes	Yes	Yes
7	How precise are the results?	Yes	Yes	Yes	Yes	Yes	Yes	Yes	Yes
8	Can the results be applied to the local population?	Yes	Yes	No	Yes	Yes	Yes	Yes	Yes
9	Were all important outcomes considered?	Yes	Yes	Yes	Yes	Yes	Yes	Yes	Yes
10	Are the benefits worth the harms and costs?	Yes	Yes	Yes	Yes	Yes	Yes	Yes	Yes

## DISCUSSION

### Critical Thinking

Nurses' ability to think critically is crucial for delivering effective patient care. Various approaches, such as simulations, case studies, reflection, and RCD, have been employed to enhance nurses' critical thinking skills. Many studies focus on RCD, for instance, Ardian's research which adopts the Graham Gibss Cycle technique to measure critical thinking dimensions in the form of thinking engagement, cognitive maturity, and innovativeness [16]. The findings from this study are consistent with previous research by Dalheim, Harthug, Nilsen, and Nortvedt, which highlighted that nurses often rely on their experiences rather than incorporating research findings into their practice [25]. This experience is very identical to the RCD process. Experiences are also able to influence critical thinking skills. Moreover, nurses' critical thinking skills tend to improve with increasing experience, which also contributes to their professionalism in decision-making [18, 24]. Another study by Ibrahim revealed the correlation between RCD training and improving critical thinking skills and interpersonal communication among nurse apprentices in an Arabic setting [7]. RCD is grounded in clinical experience [26], and nurses' critical thinking ability is closely associated with making sound decisions [27].

### Conceptual skill

Skills in providing nursing care to patients are needed, especially when collaborating with other health workers. Koopmans argues that skilled nurses contribute to the client's quality of life, job satisfaction, and quality of care provided [28] and have a strong sense of high affection [29]. Furthermore, another research states that nurses with competency and expertise certificates will contribute to good clinical outcomes and have little or no impact on patient mortality [30]. This is also in line with Madison's opinion, which states that reflective practice contributes to skills discretion, self-efficacy, and autonomy [31]. The RCD concept in Timmins' research also explored the benefits of reflective practice not only for action skills but also their relational skills [32]. These skills are intended not only for colleagues but also for all health professionals. Jane's research also supports this finding [33]. In specific cases, RCD proved particularly beneficial for nurses' skills. It serves as an effective approach to address errors that may arise from negligence or limited skills. Through the process of RCD, nurses can critically reflect on their actions, identify areas of improvement, and develop strategies to prevent the recurrence of similar errors in the future [8].

### Knowledge

Providing optimal healthcare services is closely linked to employing trained and knowledgeable personnel within hospitals [34]. This is also reflected in patient satisfaction. This is in line with Aiken *et al*. [35], indicating that hospitals with skilled nurses have significant benefits for patients compared to those without. RCD is a method that has been proven to increase nurses' knowledge [11], supporting Dawber's research, which highlights how RCD facilitates nurses in gaining clinical insight [36]. The reflective nature of RCD assists nurses in better understanding their practice and its impact on patients [37]. This finding is consistent with Al-Osaimi's research, which demonstrates that reflective practice among nursing students increases their knowledge [38-41].

Some of the limitations of this review include the restriction to papers published in the English language and the focus on articles conducted in clinical settings. These limitations should be acknowledged as they may influence the generalizability and scope of the findings.

## CONCLUSION

The findings of this review underscore the significance of reflective practice in enhancing the professionalism of nurses in hospital settings. Reflective Case Discussion (RCD) serves as a valuable tool for discussing and refining Standard Operating Procedures (SOPs). It not only contributes to improved teamwork and mutual respect among nurses but also provides a platform for sharing experiences and minimizing the occurrence of repeated mistakes.
